# CMC: Cancer miRNA Census – a list of cancer-related miRNA genes

**DOI:** 10.1093/nar/gkae017

**Published:** 2024-01-23

**Authors:** Malwina Suszynska, Magdalena Machowska, Eliza Fraszczyk, Maciej Michalczyk, Anna Philips, Paulina Galka-Marciniak, Piotr Kozlowski

**Affiliations:** Department of Molecular Genetics, Institute of Bioorganic Chemistry, Polish Academy of Sciences, Poznan, 61-704, Poland; Department of Molecular Genetics, Institute of Bioorganic Chemistry, Polish Academy of Sciences, Poznan, 61-704, Poland; Department of Molecular Genetics, Institute of Bioorganic Chemistry, Polish Academy of Sciences, Poznan, 61-704, Poland; Laboratory of Bioinformatics, Institute of Bioorganic Chemistry, Polish Academy of Sciences, Poznan, Poland; Laboratory of Bioinformatics, Institute of Bioorganic Chemistry, Polish Academy of Sciences, Poznan, Poland; Department of Molecular Genetics, Institute of Bioorganic Chemistry, Polish Academy of Sciences, Poznan, 61-704, Poland; Department of Molecular Genetics, Institute of Bioorganic Chemistry, Polish Academy of Sciences, Poznan, 61-704, Poland

## Abstract

A growing body of evidence indicates an important role of miRNAs in cancer; however, there is no definitive, convenient-to-use list of cancer-related miRNAs or miRNA genes that may serve as a reference for analyses of miRNAs in cancer. To this end, we created a list of 165 cancer-related miRNA genes called the Cancer miRNA Census (CMC). The list is based on a score, built on various types of functional and genetic evidence for the role of particular miRNAs in cancer, e.g. miRNA–cancer associations reported in databases, associations of miRNAs with cancer hallmarks, or signals of positive selection of genetic alterations in cancer. The presence of well-recognized cancer-related miRNA genes, such as *MIR21*, *MIR155*, *MIR15A*, *MIR17* or *MIRLET7s*, at the top of the CMC ranking directly confirms the accuracy and robustness of the list. Additionally, to verify and indicate the reliability of CMC, we performed a validation of criteria used to build CMC, comparison of CMC with various cancer data (publications and databases), and enrichment analyses of biological pathways and processes such as Gene Ontology or DisGeNET. All validation steps showed a strong association of CMC with cancer/cancer-related processes confirming its usefulness as a reference list of miRNA genes associated with cancer.

## Graphical Abstract

**Graphical Abstract:**
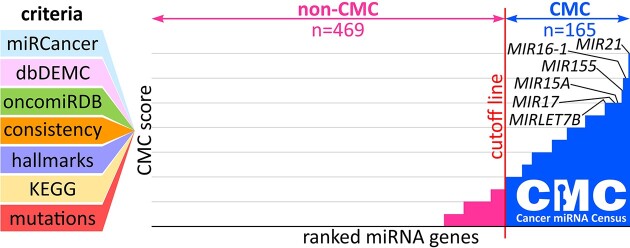

## Introduction

miRNAs are a class of small (18–24nt long), single-stranded noncoding RNA molecules that initiate translation repression and/or mRNA deadenylation, decapping, and degradation by complementary interaction with target sequences usually located in the 3′UTRs of target mRNAs; thus, miRNAs post-transcriptionally regulate (usually downregulate) the expression of most protein-coding genes (1,2). A large body of evidence indicates that miRNAs play an important role in cancer, and many upregulated and downregulated miRNAs are involved in the regulation of different processes in cancer. Among the most well-recognized miRNAs, acting either as oncogenes or tumor suppressors are the let-7 (*MIRLET7*) family, *miR-17-92a-1* cluster (known also as OncomiR-1 or according to HUGO nomenclature *MIR17HG*), miR-21, miR-205, and miR-15a (3,4).

Large cancer-genome projects such as The Cancer Genome Atlas (TCGA) (5,6) or International Cancer Genome Consortium (ICGC) (7) and especially recent whole-genome sequencing efforts such as The Pan-Cancer Analysis of Whole Genomes (PCAWG) (8) increase the potential for identifying cancer-related mutations or cancer-driver genetic elements in noncoding parts of the genome, including genes of miRNAs and other classes of noncoding RNAs. Appropriate reference lists of cancer-related noncoding elements would facilitate establishing a link between identified mutations in noncoding regions and cancer or optimizing statistical approaches for such identifications (9–11).

There are two main databases of miRNAs that we will refer to in the study: miRBase (12,13), the most commonly used, usually considered the reference list of miRNAs; and MirGeneDB (14,15), a manually curated database, based on very stringent criteria of miRNA precursor structure, miRNA detectability in a wide panel of tissues, and miRNA evolutionary conservation. It should be noted, however, that, a substantial number of approximately 2000 annotated human miRNA genes are not fully validated, i.e. are rarely expressed on a detectable level (16–18), and in some cases, do not meet the basic criteria for canonical miRNA precursor structure and processing (19). The inclusion of such miRNAs in any global analysis would result in obvious biases, e.g. they are unlikely to be detected in differential expression analyses. Also, such miRNA genes may be located in genomic regions that undergo different mutagenic and/or DNA repair processes, for example, they may not undergo effective transcription-dependent DNA repair (20,21).

Despite the substantial interest in and large number of studies investigating the role of miRNAs in cancer and even though several miRNAs (including those mentioned above) are well recognized as crucial regulators of cancer-related processes, there is no comprehensive list of miRNA genes attributed to cancer. Although there are numerous review articles on different aspects of miRNAs in cancer and several well-recognized databases of information on different types of associations of miRNAs with cancer (e.g. miRCancer (22), database of Differentially Expressed MiRNAs in human Cancers (dbDEMC) (23), or oncomiRDB (24)), none of them provides a definitive reference list of cancer-related miRNAs or miRNA genes (as distinct from non-cancer-related ones). Additionally, distinguishing cancer-related from non-cancer-related miRNAs solely based on a literature occurrence would be almost impossible and would likely result in the classification of nearly all miRNA genes, even those questionable ones, to the cancer-related pool. It is also worth noting that many studies of miRNAs in cancer are of low reliability which has resulted that many such studies have been recently retracted from different journals (e.g. see the list of miRNA-related articles retracted after rigorous editorial re-evaluation from *Journal of Cellular Biochemistry*: https://pubmed.ncbi.nlm.nih.gov/?term=%22J+Cell+Biochem%22%5Bjour%5D+retraction+mirna&sort=date&size=200).

Analysis of protein-coding genes in cancer has been greatly facilitated by the Cancer Gene Census (CGC; developed based on the Catalogue Of Somatic Mutations In Cancer (COSMIC), an initiative of the Wellcome Sanger Institute), the expert-curated list of genes implicated in cancer whose mutations/aberrations drive the development of cancer (8,25,26). CGC is commonly used as a reference in basic research and clinically related studies of human cancers. The lack of similar reference lists for noncoding genetic elements has seriously hampered analyses of the role of these elements in cancer, especially the identification of cancer-driver mutations in these regions. Few such mutations have been detected so far (11,27–34). The first and only attempt to date to create similar resources for noncoding elements is a list of cancer-associated long noncoding RNAs (lncRNAs) called the Cancer lncRNA Census (CLC) (analogous to CGC) (35). Such datasets may serve as objective reference lists for validating genome-wide experimental results, e.g. cancer-driver identification, for prioritizing genes/genetic elements identified in cancer analyses for their further analyses, or for differential expression analyses.

To facilitate research on miRNAs in cancer, we compiled a list of cancer-related miRNA genes called the Cancer miRNA Census (CMC) (analogous to CGC and CLC). The list is based on several criteria, including miRNA–cancer associations indicated in 3 cancer-related databases, i.e. miRCancer (22), dbDEMC (23), and oncomiRDB (24), as well as other evidence supporting the role of miRNAs in cancer such as miRNAs associated with cancer hallmarks (36), the Kyoto Encyclopedia of Genes and Genomes (KEGG) ‘MicroRNAs in cancer’ pathway, and well-documented genetic evidence for positive selection of cancer somatic alterations, resulting in enrichment of mutations in miRNA genes (11,27–33). Like CGC and CLC, CMC could serve as a valuable, standard reference for the validation of results of various large-scale experimental and computational analyses of miRNAs or miRNA genes in cancer, for the optimization of analytical procedures for such studies, and for the prioritization of miRNAs or miRNA genes detected in whole miRNome or whole-genome studies for further functional and/or clinical characterization. The CMC miRNA genes and their characteristics are accessible through the CMC Cancer miRNA Census portal.

## Methods

### miRNA databases and nomenclature

For the specification of miRNA genes, we downloaded IDs of all (*n* = 1917) human miRNA precursors/genes annotated in miRBase v.22.1 (www.mirbase.org/ May 2022), and 507 miRNA genes annotated in MirGeneDB v.2.1 (www.mirgenedb.org/ May 2022). We did not include seven miRNA genes annotated in MirGeneDB that did not have counterparts in miRBase. Additionally, each miRNA gene was annotated with a gene ID according to the HUGO Gene Nomenclature Committee at the European Bioinformatics Institute (www.genenames.org/), miRNA ID according to miRBase, information about the precursor arm (5p or 3p) from which the mature miRNA is primarily generated (as defined before (11,37)), and information about the confidence of annotation in miRBase (if HIGH) (Supplementary Table S1). As a substantial number of annotated miRNA genes are not fully validated (see Introduction), thus for the selection of cancer-related CMC miRNA genes, we used only a list of convincingly validated miRNA genes, hereafter used as the background list of miRNA genes (*n* = 634). The miRNA genes were classified as the background list based on their designation as ‘high confidence’ in miRBase (*n* = 505) (12,13) and/or annotation in MirGeneDB (*n* = 507) (14,15). As shown in Supplementary Figure S1, both groups of miRNA genes strongly overlap with each other (compared to an overlap expected by chance; fold enrichment (FE) = 2.8, *P*= 1.2E−171, hypergeometric probability test), which further confirmed the robustness of the designation of background miRNA genes.

It is important to note that all analyses performed in this study were based on miRNA genes/loci (with the use of unequivocal HUGO gene IDs) and not on miRNAs themselves. This strategy allowed us to prevent inconsistencies resulting from the fact that in some situations, the same mature miRNA, e.g. miR-1 (that may also be designated as miR-1-3p), may be coded by different genes (precursors), here *MIR1-1* (miR-1-1) and *MIR1-2* (miR-1-2) (Supplementary Table S1).

### Scoring and selection of cancer-related miRNA genes for CMC

Each miRNA gene was scored according to the following criteria: the number of miRNA-cancer associations reported in three databases, namely, [criterion I] miRCancer (http://mircancer.ecu.edu) (22), [criterion II] dbDEMC v3.0 (https://www.biosino.org/dbDEMC/index) (23), and [criterion III] oncomiRDB (http://lifeome.net/database/oncomirdb/) (24) (separately for each database, 1 point was assigned for the top 10% and 0.5 points for the remaining top 25% with the highest number of associations; borderline miRNA genes with the same number of associations were ranked upwards; Supplementary Table S2); [criterion IV] the consistency of the direction of associations with either increased level/oncogenic properties or decreased level/tumor suppressor properties (for miRCancer and dbDEMC/oncomiRDB, respectively) observed both within each database (≥75% associations in the same direction, not including associations without determined direction, annotated as NPA in oncomiRDB) and between all the databases (1 point; Supplementary Table S2); [criterion V] the role of miRNA in regulating (positively or negatively) at least one cancer hallmark, based on Supplemntary Information deposited in GitHub (36) (1 point; Supplementary Table S3); [criterion VI] the presence of miRNA in the KEGG pathway map05206: ‘MicroRNAs in cancer’ (www.genome.jp/entry/pathway+hsa05206) (1 point; Supplementary Table S4); and [criterion VII] genetic evidence for positive selection of genetic alterations in cancer (enrichment of somatic mutations) (11,27–33) (1 point; Supplementary Table S4). [Re criteria I-III] Information on the number of miRNA–cancer associations was retrieved from the databases as of May 2022 from miRCancer and March 2023 from dbDEMC and oncomiRDB. In brief, miRCancer, last updated June 2020, is based on a text-mining strategy of PubMed records (with 75 different rules) together with manual verification of the detected associations and cumulatively consists of 9006 associations (22); dbDEMC, last updated June 2021, is based on differential expression analyses of publicly available datasets of high-throughput methods such as microarray or miRNA-seq (and in some cases of low-throughput methods such as RT-qPCR or northern blot) and cumulatively consists of 53 185 associations for human miRNAs taking into account the ‘cancer vs. normal’ category based on high-throughput methods (23); and oncomiRDB, last updated January 2014, is based on manual PubMed literature searches, focusing only on experimentally verified ‘OncomiRs’ defined as miRNAs regulating cancer hallmarks, e.g. proliferation, apoptosis, migration, or targeting oncogenic and tumor-suppressor genes by miRNAs, and it cumulatively consists of 2259 associations (24). From dbDEMC, only associations of the design ‘cancer versus normal’ for human miRNAs identified by high-throughput methods were taken into account. If the association applied to a miRNA that can be expressed from more than one miRNA gene, it was counted separately for each of the genes (e.g. associations of miR-1 were assigned both to *MIR1-1* and *MIR1-2*). In cases, where a particular gene had associations for both miR-5p and miR-3p, the number of associations was counted cumulatively and attributed to the particular gene (e.g. miR-125a-5p and miR-125a-3p to *MIR125A* and miR-140-5p and miR-140-3p to *MIR140*). Associations were counted to the adequate gene also in cases where no strand (5p or 3p) was indicated (e.g. miR-100 to *MIR100*). [Re criterion V] The miRNAs regulating cancer hallmarks were identified based on recently published pancancer analysis of transcriptomic, methylation, and mutation data of >7000 cancer samples from TCGA utilizing penalized regression techniques (36). [Re criterion VII] miRNA genes with positive cancer selection signals (mutation enrichment) were identified based on a manual literature search performed by us and were limited to only a few of the most convincingly demonstrated miRNA genes (11,27–33).

### CMC validation analyses

For pairwise comparison between the numbers of associations reported by the databases, the Spearman correlation coefficient (rho) and appropriate p-value were calculated. Leave-one-out analysis was performed for each criterion building the CMC score. Each criterion was one by one removed from the score, a new list of CMC miRNA genes was selected (new CMC selected based on the same criteria except for the left/tested criterion; with the same predefined cutoff ≥2 points), and then FE was calculated comparing the observed versus expected numbers of the miRNA genes, scored by the left/tested criterion, in the new CMC. In a similar way, FE values were calculated for the other groups of miRNA genes compared to CMC. These groups included miRNAs associated with individual cancer hallmark/datasets (36) (Supplementary Table S3); genes of miRNAs defined as differentially expressed in specific cancer types in research articles (listed in Supplementary Data S5 from Hamilton *et al.* (38), Table 2 from Vishnubalaji *et al.* (39), Supplementary Table S1 from Oh *et al.* (40), Supplementary Tables 4A and 4B from Koshizuka *et al.* (41), Figure 2 from Wach *et al.* (42), Table 2 from Kozubek et al. (43), Supplementary Table S2 from Krasniqi et al. (44), Supplementary Table S4 from Mancikova *et al.* (45)); miRNAs specified as attributed to specific cancer types in review articles (listed in Tables 1 and 2 from Yoshino *et al.* (46), Tables 1 and 2 from van Schooneveld *et al.* (47), Table 1 from Zhu *et al.* (48), Tables 2 and 3 from Yonemori *et al.* (49), Tables 2 and 3 from Goto *et al.* (50)); miRNAs differentially expressed at the level of nominal *P*< 0.0001 in TCGA cancer types, as specified in the OncomiR database (https://www.oncomir.org) (51); and miRNA genes showing the impact on cell growth/fitness in CRISPR-based miRNA genes knock-out screenings in three cancer cell lines, i.e. cervical (Supplementary Table S4 from Kurata *et al.* (52)), gastric (Supplementary Table S8 from Kurata *et al.* (52)), and myeloid leukemia (S2 Table from Wallace *et al.* (53)) (Supplementary Tables S5 and S6).

**Table 1.** CMC miRNA genes (*n* = 165) with points from each of the criteria [I–VII] and CMC score

CMC miRNA genes	[I]	[II]	[III]	[IV]	[V]	[VI]	[VII]	CMC score	O/TS	CMC miRNA genes	[I]	[II]	[III]	[IV]	[V]	[VI]	[VII]	CMC score	O/TS
*MIR21*	1	1	1	1	1	1	1	**7**	O	*MIR122*	0.5		1			1	1	**3.5**	
*MIR101-1*	1	1	1	1	1	1		**6**	TS	*MIR128-1*	0.5	0.5	0.5		1	1		**3.5**	
*MIR101-2*	1	1	1	1	1	1		**6**	TS	*MIR128-2*	0.5	0.5	0.5		1	1		**3.5**	
*MIR143*	1	1	1	1	1	1		**6**	TS	*MIR146A*	0.5		1		1	1		**3.5**	
*MIR145*	1	1	1	1	1	1		**6**	TS	*MIR192*	0.5	0.5	0.5		1	1		**3.5**	
*MIR16-1*	1	1	1		1	1	1	**6**		*MIR194-1*	0.5	0.5	0.5		1	1		**3.5**	
*MIR26A1*	1	1	1	1	1	1		**6**	TS	*MIR194-2*	0.5	0.5	0.5		1	1		**3.5**	
*MIR26A2*	1	1	1	1	1	1		**6**	TS	*MIR210*	0.5	0.5	0.5		1	1		**3.5**	O
*MIR29C*	1	1	1	1	1	1		**6**	TS	*MIR224*	0.5	0.5	0.5		1	1		**3.5**	O
*MIR155*	1	0.5	1	1	1	1		**5.5**	O	*MIR23A*	0.5	0.5	0.5		1	1		**3.5**	
*MIR30A*	1	1	0.5	1	1	1		**5.5**	TS	*MIR26B*	0.5	0.5	0.5		1	1		**3.5**	
*MIR100*	0.5	1	0.5	1	1	1		**5**	TS	*MIR27B*	0.5	0.5	0.5		1	1		**3.5**	
*MIR106B*	0.5	1	0.5	1	1	1		**5**	O	*MIR301A*	0.5	0.5	0.5	1	1			**3.5**	O
*MIR10B*	1	1	1		1	1		**5**		*MIR32*	0.5	0.5	0.5		1	1		**3.5**	
*MIR126*	1	1	1		1	1		**5**		*MIR335*	0.5	0.5	0.5		1	1		**3.5**	
*MIR15A*	0.5	0.5	1		1	1	1	**5**		*MIR342*		1	0.5		1	1		**3.5**	
*MIR16-2*	1	1	1		1	1		**5**		*MIR497*	1	1	0.5	1				**3.5**	TS
*MIR17*	1	1	1		1	1		**5**	O	*MIR96*	0.5	0.5	0.5		1	1		**3.5**	O
*MIR181A1*	1	1	1		1	1		**5**		*MIRLET7E*		0.5	1		1	1		**3.5**	TS
*MIR181A2*	1	1	1		1	1		**5**		*MIRLET7I*		0.5	1		1	1		**3.5**	
*MIR199A1*	1	1	1		1	1		**5**		*MIR103A1*		0.5	0.5		1	1		**3**	O
*MIR199A2*	1	1	1		1	1		**5**		*MIR103A2*		0.5	0.5		1	1		**3**	O
*MIR19A*	1	0.5	0.5	1	1	1		**5**	O	*MIR107*	0.5		0.5		1	1		**3**	
*MIR205*	1		1		1	1	1	**5**		*MIR10A*	0.5		0.5		1	1		**3**	
*MIR221*	1	1	1		1	1		**5**		*MIR124-1*	1		1			1		**3**	
*MIR29B1*	1	1	1		1	1		**5**		*MIR124-2*	1		1			1		**3**	
*MIR29B2*	1	1	1		1	1		**5**		*MIR124-3*	1		1			1		**3**	
*MIR30E*	0.5	1	0.5	1	1	1		**5**		*MIR130B*	0.5	1	0.5		1			**3**	
*MIR31*	1	1	1		1	1		**5**		*MIR133B*	0.5		0.5	1		1		**3**	TS
*MIR34A*	1	1	1		1	1		**5**		*MIR144*	0.5	1	0.5		1			**3**	TS
*MIR99A*	0.5	1	0.5	1	1	1		**5**	TS	*MIR148A*	1	0.5	0.5		1			**3**	
*MIRLET7B*	0.5	0.5	1	1	1	1		**5**	TS	*MIR152*	0.5		0.5		1	1		**3**	
*MIRLET7C*	0.5	0.5	1	1	1	1		**5**	TS	*MIR193B*		0.5	0.5		1	1		**3**	
*MIR133A1*	1	0.5	1	1		1		**4.5**	TS	*MIR199B*		0.5	0.5		1	1		**3**	
*MIR133A2*	1	0.5	1	1		1		**4.5**	TS	*MIR204*	1	0.5	0.5	1				**3**	TS
*MIR141*	1	1	0.5		1	1		**4.5**		*MIR218-1*	1	0.5	0.5		1			**3**	TS
*MIR183*	1	1	0.5		1	1		**4.5**	O	*MIR218-2*	1	0.5	0.5		1			**3**	TS
*MIR195*	1	1	0.5	1		1		**4.5**	TS	*MIR23B*	0.5		0.5		1	1		**3**	
*MIR200A*	0.5	1	1		1	1		**4.5**		*MIR24-1*	0.5	1	0.5		1			**3**	
*MIR200B*	0.5	1	1		1	1		**4.5**		*MIR24-2*	0.5	1	0.5		1			**3**	
*MIR200C*	1	0.5	1		1	1		**4.5**		*MIR28*		1			1	1		**3**	
*MIR203A*	1	0.5	1		1	1		**4.5**		*MIR30B*		0.5	0.5		1	1		**3**	
*MIR20A*	1	1	0.5		1	1		**4.5**	O	*MIR30D*		0.5	0.5		1	1		**3**	
*MIR222*	1	0.5	1		1	1		**4.5**	O	*MIR324*		1			1	1		**3**	
*MIR29A*	1	0.5	1		1	1		**4.5**		*MIR378A*	0.5	1	0.5		1			**3**	
*MIR30C1*		1	0.5	1	1	1		**4.5**	TS	*MIR93*	0.5	1	0.5		1			**3**	O
*MIR30C2*		1	0.5	1	1	1		**4.5**	TS	*MIRLET7G*			1		1	1		**3**	
*MIR451A*	0.5	0.5	0.5	1	1	1		**4.5**	TS	*MIR129-1*	0.5	0.5	0.5			1		**2.5**	TS
*MIR7-1*	0.5	1	1		1	1		**4.5**		*MIR129-2*	0.5	0.5	0.5			1		**2.5**	TS
*MIR7-2*	0.5	1	1		1	1		**4.5**		*MIR130A*	0.5	0.5	0.5		1			**2.5**	
*MIR7-3*	0.5	1	1		1	1		**4.5**		*MIR135B*	0.5	0.5	0.5			1		**2.5**	O
*MIR9-1*	1	0.5	1		1	1		**4.5**		*MIR137*	1		0.5			1		**2.5**	
*MIR9-2*	1	0.5	1		1	1		**4.5**		*MIR146B*	0.5	0.5	0.5		1			**2.5**	
*MIR92A1*	1	1	0.5		1	1		**4.5**		*MIR181C*			0.5		1	1		**2.5**	
*MIR92A2*	1	1	0.5		1	1		**4.5**		*MIR193A*		1	0.5		1			**2.5**	
*MIR9-3*	1	0.5	1		1	1		**4.5**		*MIR22*	0.5	0.5	0.5		1			**2.5**	
*MIRLET7A1*	1	0.5	1		1	1		**4.5**		*MIR34B*	0.5		1			1		**2.5**	
*MIRLET7A2*	1	0.5	1		1	1		**4.5**		*MIR34C*	0.5		1			1		**2.5**	
*MIRLET7A3*	1	0.5	1		1	1		**4.5**		*MIR423*		0.5			1	1		**2.5**	
*MIR1-1*	0.5	0.5	1	1		1		**4**	TS	*MIR455*	0.5	1			1			**2.5**	
*MIR1-2*	0.5	0.5	1	1		1		**4**	TS	*MIR106A*	0.5	0.5			1			**2**	
*MIR125A*	0.5	1	0.5		1	1		**4**	TS	*MIR132*	0.5	0.5			1			**2**	
*MIR125B1*	1	1	1			1		**4**		*MIR135A1*	0.5		0.5			1		**2**	
*MIR125B2*	1	1	1			1		**4**		*MIR135A2*	0.5		0.5			1		**2**	
*MIR139*	0.5	1	0.5	1	1			**4**	TS	*MIR138-1*	1	0.5	0.5					**2**	TS
*MIR140*	0.5	1	0.5	1	1			**4**	TS	*MIR138-2*	1	0.5	0.5					**2**	TS
*MIR142*	0.5	1	0.5		1		1	**4**		*MIR148B*	0.5		0.5		1			**2**	
*MIR150*	1	0.5	0.5		1	1		**4**		*MIR149*		0.5	0.5		1			**2**	
*MIR15B*	0.5	1	0.5		1	1		**4**		*MIR206*	0.5		0.5			1		**2**	TS
*MIR181B1*	0.5	0.5	1		1	1		**4**		*MIR215*	0.5		0.5			1		**2**	
*MIR181B2*	0.5	0.5	1		1	1		**4**		*MIR320A*	0.5		0.5		1			**2**	
*MIR182*	1	1	1		1			**4**	O	*MIR331*		0.5	0.5			1		**2**	
*MIR18A*	0.5	1	0.5		1	1		**4**	O	*MIR345*					1	1		**2**	
*MIR19B1*	0.5	1	0.5		1	1		**4**		*MIR373*	0.5		0.5			1		**2**	
*MIR19B2*	0.5	1	0.5		1	1		**4**		*MIR449A*	0.5		0.5			1		**2**	
*MIR214*	1	0.5	0.5		1	1		**4**		*MIR483*		0.5	0.5			1		**2**	
*MIR223*	1	0.5	0.5		1	1		**4**		*MIR486-1*	0.5	0.5			1			**2**	TS
*MIR25*	0.5	1	0.5		1	1		**4**	O	*MIR486-2*	0.5	0.5			1			**2**	TS
*MIR27A*	1	0.5	0.5		1	1		**4**		*MIR494*	0.5		0.5			1		**2**	
*MIR375*	1		1		1	1		**4**		*MIR625*					1	1		**2**	
*MIRLET7D*	0.5	0.5	1		1	1		**4**		*MIR98*	0.5		0.5		1			**2**	
*MIRLET7F1*	0.5	0.5	1		1	1		**4**		*MIR99B*		0.5	0.5		1			**2**	
*MIRLET7F2*	0.5	0.5	1		1	1		**4**											

O – oncogene, TS - tumor suppressor.

### Gene set fold enrichment analysis

miRNA targets for miRNAs derived from precursors coded by all 634 background miRNA genes (expressed from 3p, 5p or both arms of the miRNA precursor, as specified in Supplementary Table S7, column ‘predominantly expressed miRNA (miRNA-strand balance)’) were acquired from miRTarBase v9.0 (https://mirtarbase.cuhk.edu.cn), a well-recognized database of experimentally validated miRNA targets (54). To avoid saturation of the analysis resulting from a very large number of targets assigned to many miRNAs in miRTarBase and to reduce the bias resulting from the large variability in the number of assigned targets (from a few to >100), only the two top-validated targets were assigned for each miRNA. The validation of a particular target was determined by the multiplication of the number of methods used to validate the target and the number of papers reporting the target. If more than one target had the same score, we further took into account the number of ‘strong evidence’ i.e. low-throughput methods, and if still more than one target had the same ranking, targets were selected in alphabetic order. A total of 181 and 576 unique target genes were assigned to CMC and non-CMC, respectively (Supplementary Table S7), and used for enrichment analysis in terms determined in Gene Ontology Biological Process (BP; GOTERM_BP_DIRECT); DisGeNET—a database of gene-disease associations; Reactome pathways, KEGG pathways and Molecular Signatures Database (MSigDB) hallmarks (55–57). The analysis was performed with the use of The Database for Annotation, Visualization and Integrated Discovery (DAVID) (58–60) or ShinyGO (61) (for MSigDB) presenting the associations with FE and false discovery rate (FDR) adjusted *q*-values (*q* < 0.05 were considered significant) (Supplementary Table S8). Additionally, both lists of CMC and non-CMC target genes were compared with CGC, a list of cancer-driving protein-coding genes (26).

### Designation of CMC miRNA genes as potential oncogenes and tumor suppressors

Based on the collected data, CMC miRNA genes fulfilling the following criteria were classified, respectively, as potential oncogenes or potential tumor suppressors: (i) determined in at least two of the three databases (criteria I–III) as consistently associated (≥75% associations in the same direction) with increased level/oncogenic properties or decreased level/tumor suppressor properties; (ii) if the direction of associations was consistent in two databases, it could not be the opposite in the third database (it may be undetermined); (iii) the direction of associations in the databases had to be consistent with the direction of ≥75% significant changes in miRNA level (respectively, increased or decreased) tested in 16 TCGA cancer types (as specified in the OncomiR database (51) at the level of nominal *P*<0.0001, and indicated in Supplementary Table S9). To assess the association of oncogene/tumor suppressor classification with the expression level changes reported in different cancer types in research and review articles (38–50) and with the direction of association with cancer hallmarks (36) Fisher's exact test was used.

### Statistics and resources

Unless stated otherwise, statistical analyses were performed and graphs were generated using MedCalc, Real Statistics Resource Pack software (www.real-statistics.com), or GraphPad. FE was calculated as a ratio of observed versus expected values in CMC and tested with Fisher's exact test. Unless stated otherwise, a *P*<0.05 was considered significant. If appropriate, *P*-values were adjusted for multiple comparisons with the use of FDR, and a *q*-value < 0.05 was considered significant.

The list of all CMC miRNA genes along with their characteristics collected in the subsequent stages of the study is shown in Supplementary Table S9. Additionally, all the resources generated within the study, and lists of CMC and non-CMC miRNA genes may be searched for and/or downloaded from the CMC Cancer miRNA Census portal (http://cmc.ibch.poznan.pl/) created in Python using Flask package.

## Results

### Scoring and selection of cancer-related miRNA genes for CMC

**Figure 1.**
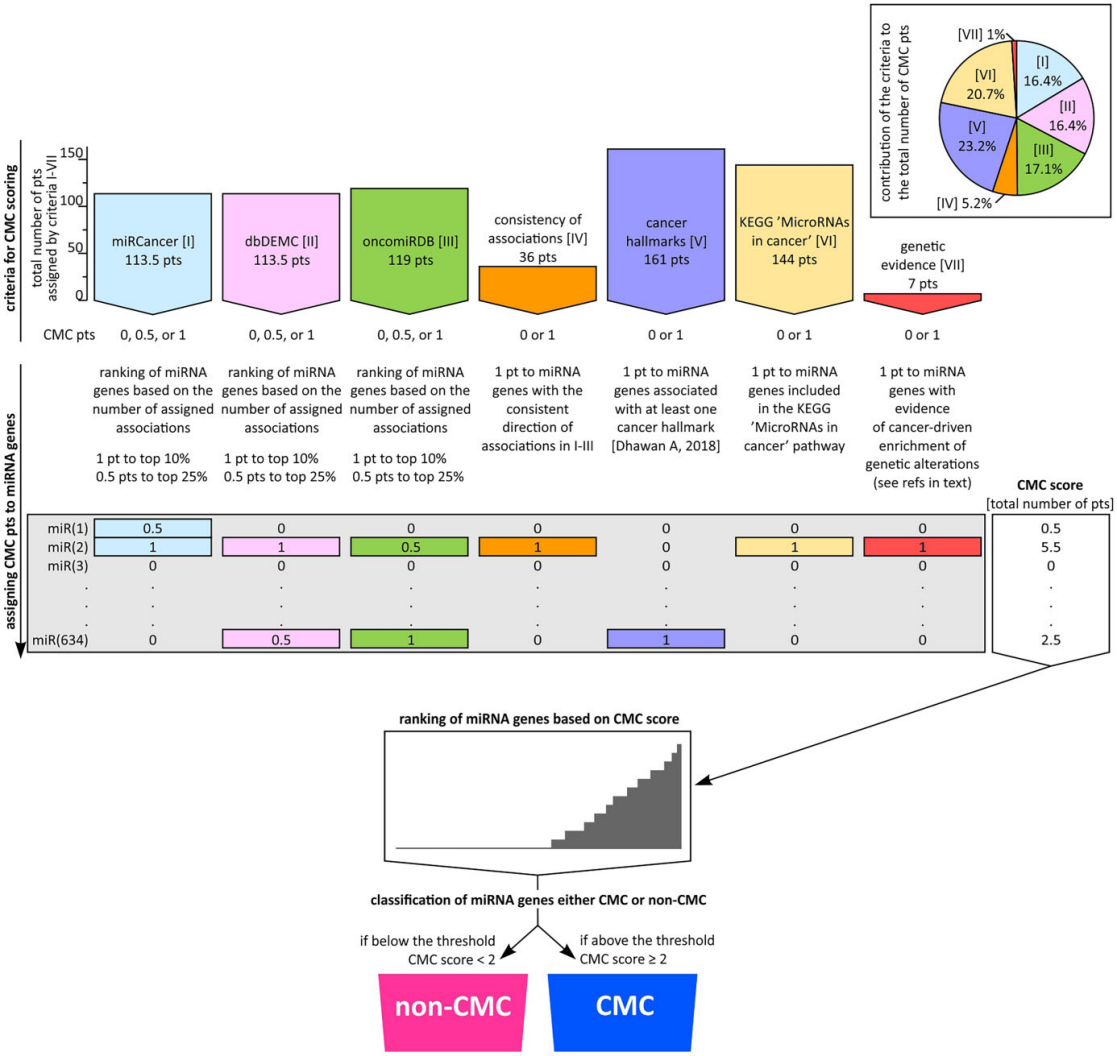
Schematic representation of the procedure used for the selection of CMC miRNA genes. The graphic shows the subsequent steps of the procedure. From the top; a graph depicting the general characteristics of criteria used for CMC scoring. The size of the bars shows the total number of points (pts) coming out (to all miRNA genes) from the particular criteria. The inset on the right depicts a pie chart showing the contribution of particular criteria to the scoring system, i.e. the proportion (%) of the points coming from the subsequent criteria to the total number of 694 pts (pts from all criteria I–VII) used for CMC scoring. In the middle; a procedure and conditions of assignment of CMC points from the particular criteria (I–VII) to particular miRNA genes (*n* = 634, note that only well-validated background miRNA genes were considered/scored; see Supplementary Figure S1). The column on the right shows the CMC scores assigned to each of the miRNA genes, i.e. the total number of points collected by particular miRNA genes from all criteria. On the bottom; based on the CMC score all miRNA genes are ranked and classified as either CMC (if above the designated threshold) or non-CMC miRNA genes.

To select cancer-related CMC miRNA genes, we developed a scoring system based on an array of conservative criteria [numbered I to VII]. Then miRNA genes with the highest scores were classified into CMC (Table 1, Figure 1, and Supplementary Table S4). It has to be noted however, that for the selection of CMC miRNA genes, we used only convincingly validated miRNA genes (*n* = 634; hereafter called background miRNA genes), selected based on validation/annotation in miRBase and MirGeneDB (for detail see Methods; Supplementary Figure S1 and Supplementary Table S1).

The main criteria were the numbers of miRNA–cancer associations reported in three databases, miRCancer [criterion I] (22), dbDEMC [criterion II] (23), and oncomiRDB [criterion III] (24). Although each of the databases gathers information about associations of miRNAs with cancer, they are highly distinctive and vary in terms of the type of collected data, method of data acquisition and curation, and size (see Methods). To ensure that the contribution (weight) of the databases to the final score was equal (Figure 1, inset), for each database separately, we ranked the miRNA genes based on the number of associations attributed to miRNAs expressed by these genes (regardless of the arm from which particular miRNAs were generated) and assigned 1 point to the top 10% and 0.5 points to the remaining top 25% of miRNA genes (Supplementary Table S2). A maximum of three points per miRNA gene was assigned based on the three databases. For miRCancer, dbDEMC, and oncomiRDB, we cumulatively assigned 113.5, 113.5 and 119 points, respectively. For miRCancer, dbDEMC, and oncomiRDB, the minimal number of associations to be included in the top 10% and top 25% of miRNA genes was 52, 162 and 19, and 22, 119 and 4, respectively.

Additionally, based on the associations reported in the miRCancer, dbDEMC and oncomiRDB databases, we assigned 1 point to 36 miRNA genes based on consistency of the direction of their associations [criterion ‘consistency of associations’ IV], with either increased expression level/oncogenic properties or decreased expression/tumor suppressor properties (Supplementary Table S2). The consistency of the association score was assigned very conservatively, based on consistency both within each database (≥75% associations in the same direction) and between the databases (the same direction in all three databases).

Further, we assigned 1 point each to miRNA genes identified as regulating at least one cancer hallmark [criterion ‘cancer hallmarks’ V] (*n* = 161; Supplementary Table S3) (36), miRNA genes listed in the ‘MicroRNAs in cancer’ KEGG pathway [criterion ‘KEGG ‘MicroRNAs in cancer’’ VI] (*n* = 144), and miRNA genes enriched in cancer somatic mutations with evidence for positive selection of genetic alterations in cancer [criterion ‘genetic evidence’ VII] (*n* = 7) (Supplementary Table S4).

The total number of points from each criterion, reflecting their weight in the final score, is shown in Figure 1. Even though some of the criteria, particularly ‘genetic evidence’ [VII], have little impact on the final score, they may have a higher weight in the future. Also, other criteria may be added to the score in the future.

**Figure 2.**
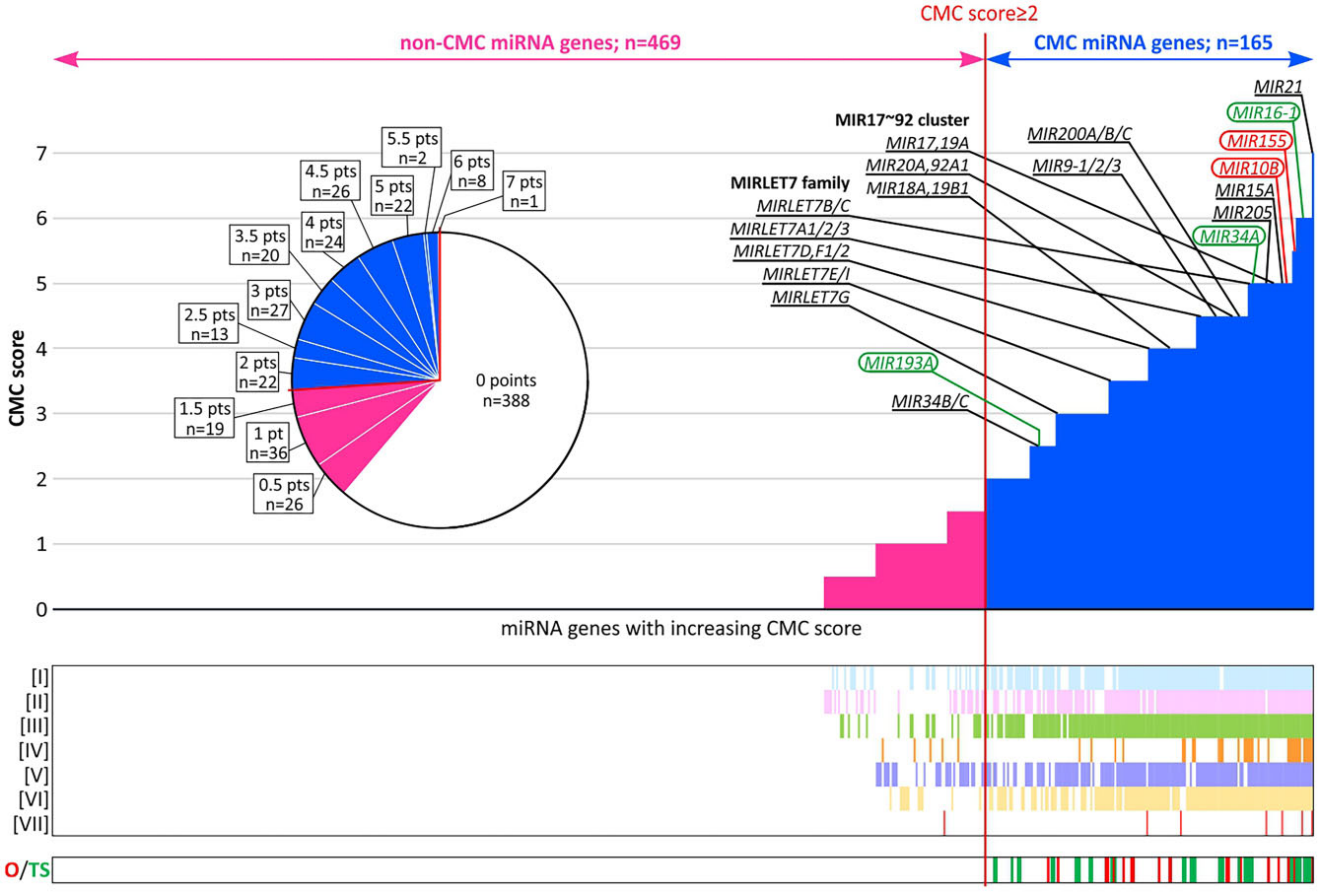
Selection of CMC cancer-related miRNA genes. A histogram showing the ranked miRNA genes (x-axis) with the increasing CMC score (y-axis). The red vertical line indicates the CMC threshold. On the left (pink part) are shown non-CMC miRNA genes, and on the right (blue part) are shown CMC miRNA genes. The top best-recognized cancer-related miRNA genes, including the genes constituting the *miR-17–92a-1* cluster and MIRLET7 family, are indicated above the graph (according to (3,4)). Of these genes, the genes of miRNAs used as therapeutics (miRNA mimics) and targets (antagomiRs) in cancer therapy clinical trials are circled in green and red, respectively (according to (96)). The pie chart shows the proportion of miRNA genes with a particular number of CMC points. The co-occurrence plots (below) indicate miRNA genes scored by particular criteria (indicated at the y-axis) and miRNA genes annotated as potential oncogenes (O) or tumor suppressors (TS).

As shown in Figure 2 and Supplementary Table S4 summarizing the distribution of the final scores, 388 of 634 miRNA genes (61.2%) have no evidence of being related to cancer (0 points). On the other hand, as noted on the graph, among the top-ranked are genes of miRNAs well known for their role in cancer, e.g. *MIR21* (with the highest score, 7 points), *MIR16-1*, *MIR155*, *MIR15A*, *MIRLET7A/B/C/D* genes, and all members of the *miR-17-92a-1* cluster (3,4). Based on the final scores, we classified 165 miRNA genes (out of 634; 26%) with a total number of points ≥2 as CMC miRNA genes (Table 1, Figure 2 and Supplementary Table S4). The threshold was set to avoid false-positive classifications of CMC miRNA genes at the expense of the potential false-negative exclusion of genuine cancer-related genes. This conservative threshold guarantees that the classified miRNA genes were, e.g. among the top 10% miRNA genes at least in two of the three databases [criteria I to III] or among the top 25% in at least two databases and additionally supported by at least one of the other criteria. The choice of the threshold was further justified by a leave-one-out analysis (see below).

### Validation of CMC

**Figure 3.**
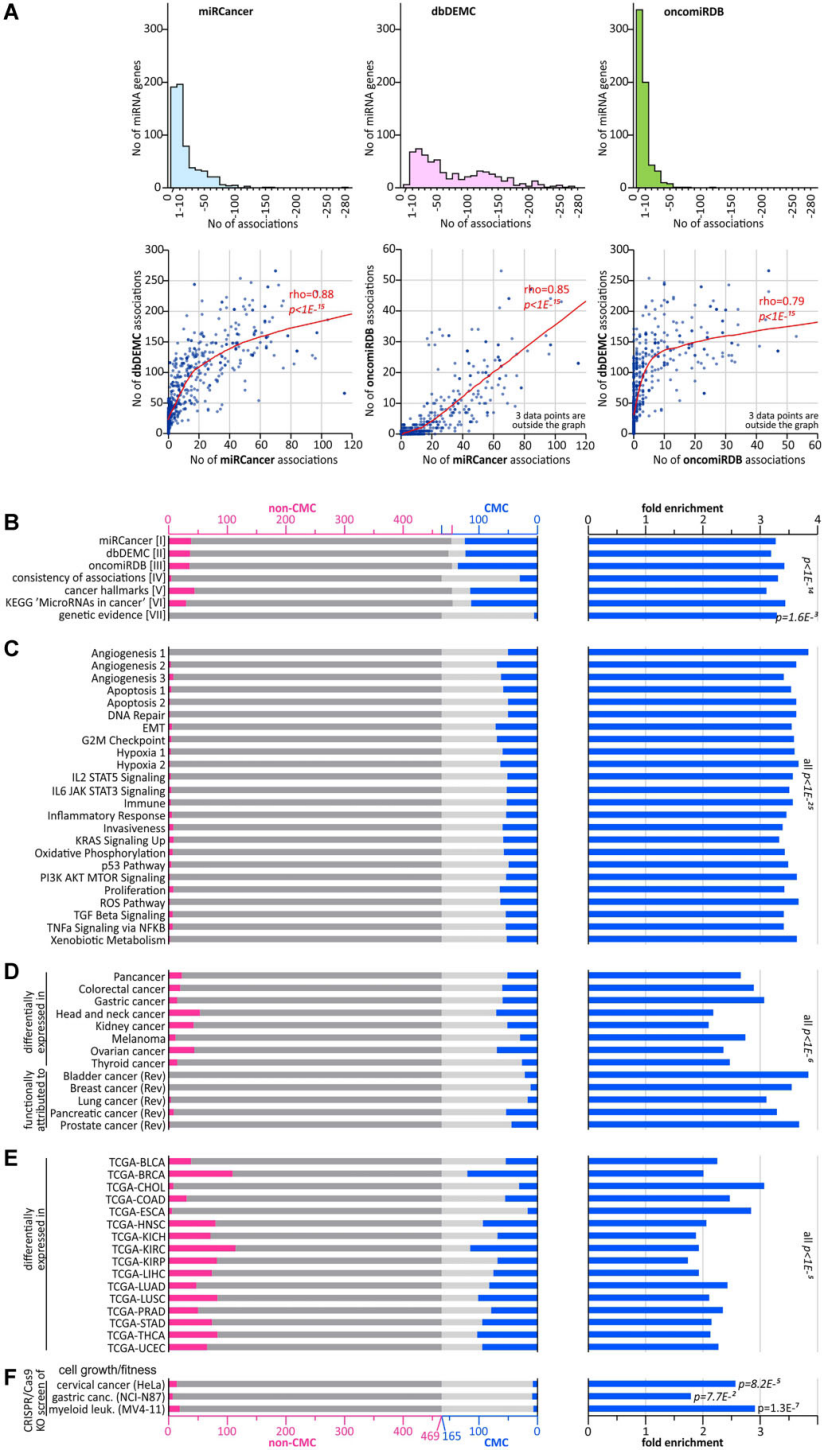
Characterization of CMC selection criteria and validation of CMC miRNA genes. (**A**) Characteristics of miRNA–cancer association data extracted from the miRCancer, dbDEMC and oncomiRDB databases [criteria I-III]. The histograms above show the distribution of the number of miRNA-cancer associations recorded in each of the databases. The bars in each of the graphs represent the number of miRNA genes (y-axis) with the following numbers of associations: 0, 1–10, 11–20, etc. (x-axis). Scatterplots below show the pairwise correlation analysis of numbers of miRNA-cancer associations attributed to particular miRNA genes (represented by the dots) in the three databases (indicated along the axes). Local regression smoothing trendline and Spearman's rank correlation coefficient (rho) are shown in red. (**B**) Results of the leave-one-out analysis of criteria I-VII. The graph [on the left] shows the occurrence of miRNA genes scored by particular criteria in non-CMC (pink bars on dark gray background) and CMC miRNA genes (blue bars on light gray background). The graph [on the right] shows FE values of miRNA genes annotated by particular criteria in a group of CMC miRNA genes (compared to expected by chance). Note that groups of miRNA annotated as CMC and non-CMC slightly differ between evaluated criteria due to the withdrawal of particular criteria from CMC scoring in the leave-one-out analysis and selection of new CMC group (see the leave-one-out procedure in Materials and Methods). (C–F) The occurrence and FE values of miRNA genes associated with 24 individual cancer hallmarks (**C**), identified as differentially expressed (in research articles) or recognized as playing a role in different cancer types (in review articles) (**D**), differentially expressed in 16 tumor types analyzed in TCGA (from OncomiR; the TCGA cancer-type abbreviations are explained in Supplementary Table S5) (**E**), and miRNA genes identified in CRISPR-based knock-out screenings as impacting cell growth/fitness (52,53) (**F**). The graphs scheme is as in (B).

To confirm the accuracy of CMC selection and the reliability of the included criteria, we performed several validation steps. First, to validate the reliability of data acquired from the databases that constitute the core of the CMC classification, we calculated the pairwise correlation between the numbers of miRNA–cancer associations attributed to particular miRNA genes based on miRCancer [I] (22), dbDEMC [II] (23), and oncomiRDB [III] (24). As shown in Figure 3A, all data were strongly correlated (Spearman correlation coefficient ranged from 0.79 to 0.88; *P <*1E-15). Although these high correlations may suggest some redundancy in the data, given that the databases are based on completely different principles, the result rather confirms the robustness and reliability of the datasets and shows their cross-validation. Additionally, based on dbDEMC, some trend of greater deviation of logFC values from 0 (value indicating no change) can be noticed for genes with higher numbers of miRNA-cancer associations (Supplementary Figure S2).

Then, to test the validity of the criteria used in the scoring system, we performed a leave-one-out analysis, subsequently withdrawing each of the seven criteria, re-selecting a new CMC (based on the same threshold, ≥2), and then calculating FE of the miRNA genes scored in the withdrawn criterion in the new CMC. All of the criteria showed significant enrichment (FE between 3.1 and 3.4; Figure 3B and Supplementary Table S6) confirming the reliability of the criteria and robustness of CMC. In addition, to verify the classification threshold (≥2) for the selected CMC miRNA genes, we repeated the leave-one-out analysis, independently of the threshold, calculating FE values, for groups of miRNA genes with a gradually increasing number of CMC points. As shown in Supplementary Figure S3, the FE values for miRNA genes with 0 points were close to 0, i.e. below values expected by chance; for miRNA genes with 1–1.5 points, the FE values were around 1, i.e. as expected by chance; and for miRNA genes with 2–2.5 points, the FE values steeply increased to ∼2.5. As expected, a further increase in CMC points in most criteria slightly increased the FE values, but the further increases were not dramatic (in most cases up to ∼3; except for criteria IV and VII with the lowest total number of points). The performed analysis further confirmed the correctness of the selected threshold of ≥ 2 for the classification of cancer-related CMC miRNA genes. The threshold clearly separates CMC miRNA genes from non-CMC miRNA genes in terms of associations with different independent criteria of cancer-related characteristics.

Among the positively enriched miRNA genes shown in the leave-one-out analysis were those associated with cancer hallmarks [criterion V; constituting any cancer hallmark] (FE = 3.1; *P*= 1.5E-59); therefore, in the next step, we checked the enrichment of miRNA genes associated with 24 individual hallmarks defined based on independent datasets (57,62–65). As shown in Figure 3C (on the left), the vast majority of miRNA genes associated with the individual hallmarks were included in CMC, and groups of miRNA genes associated with the individual hallmarks were very strongly enriched in CMC (FE between 3.3 and 3.8, all *P*< 1E-25; Figure 3C and Supplementary Table S6), even stronger than the cumulative group of miRNA genes associated with any hallmark (36).

To check the utility of CMC as a reference panel, we compared the list of miRNA genes in CMC with lists of miRNAs (i) differentially expressed in representative datasets of different cancers (*n* = 8; including pancancer analysis (38), colorectal cancer (39), gastric cancer (40), head and neck cancer (41), kidney cancer (42), melanoma (43), ovarian cancer (44) and thyroid cancer (45)) and (ii) reported in expert review articles as top-associated/related to specific cancers (*n* = 5; including bladder (46), breast (47), lung (48), pancreatic (49) and prostate cancer (50)). As shown in Figure 3D and Supplementary Table S6, all selected groups of cancer-associated miRNA genes were significantly enriched in CMC, with FE somewhat higher for miRNAs reported in the review articles (3.1 to 3.8) than differentially expressed in the individual studies/cancers (2.1–3.1). Moreover, most of the cancer-associated miRNAs fell into the CMC group compared with non-CMC (ranging from 55% up to 100%, Figure 3D).

Next, we compared CMC with groups of miRNAs differentially expressed in 16 cancer types for which appropriate data (miRNA-seq for normal and cancer samples) were available in TCGA and analysis of these data was available in the OncomiR resource (51). All groups of differentially expressed miRNAs were significantly enriched in CMC (FE 1.7–3.1, all *P*< 1E-5; Figure 3E and Supplementary Table S6). Notably, however, miRNAs assigned to particular cancer types substantially overlapped each other (Supplementary Table S5). An average of 7.7 cancer types (of 16) were associated with a particular miRNA gene in the CMC group compared to 2.2 cancer types in the non-CMC group (*t*-test, *P*< 2.28E-65).

Finally, we compared CMC with three lists of miRNA genes identified in CRISPR-based miRNA gene knock-out screenings as impacting cell growth/fitness in three different cancer cell lines (i.e. cervical, gastric and myeloid leukemia) (52,53). We found that independently on the cancer cell line and the study, miRNA genes identified as associated with cell growth/fitness were enriched in CMC (FE 1.8–2.9, Figure 3F and Supplementary Table S6).

### Gene set fold enrichment analysis and association of CMC target genes with CGC

To identify processes and pathways associated with CMC, with the use of miRTarBase (54), we assigned up to two (if available) top experimentally validated target genes for each (mature 5p and/or 3p) miRNA generated from CMC miRNA genes. For comparison, in the same way, we selected target genes associated with non-CMC miRNA genes. In total, 181 and 576 unique target genes were assigned to 175 miRNAs and 598 miRNAs (5p or 3p) expressed from CMC and non-CMC miRNA genes, respectively (Supplementary Table S7). Then, we performed gene set fold enrichment analysis of Gene Ontology BP, DisGeNET, Reactome pathways, KEGG pathways terms, and MSigDB hallmarks.

**Figure 4.**
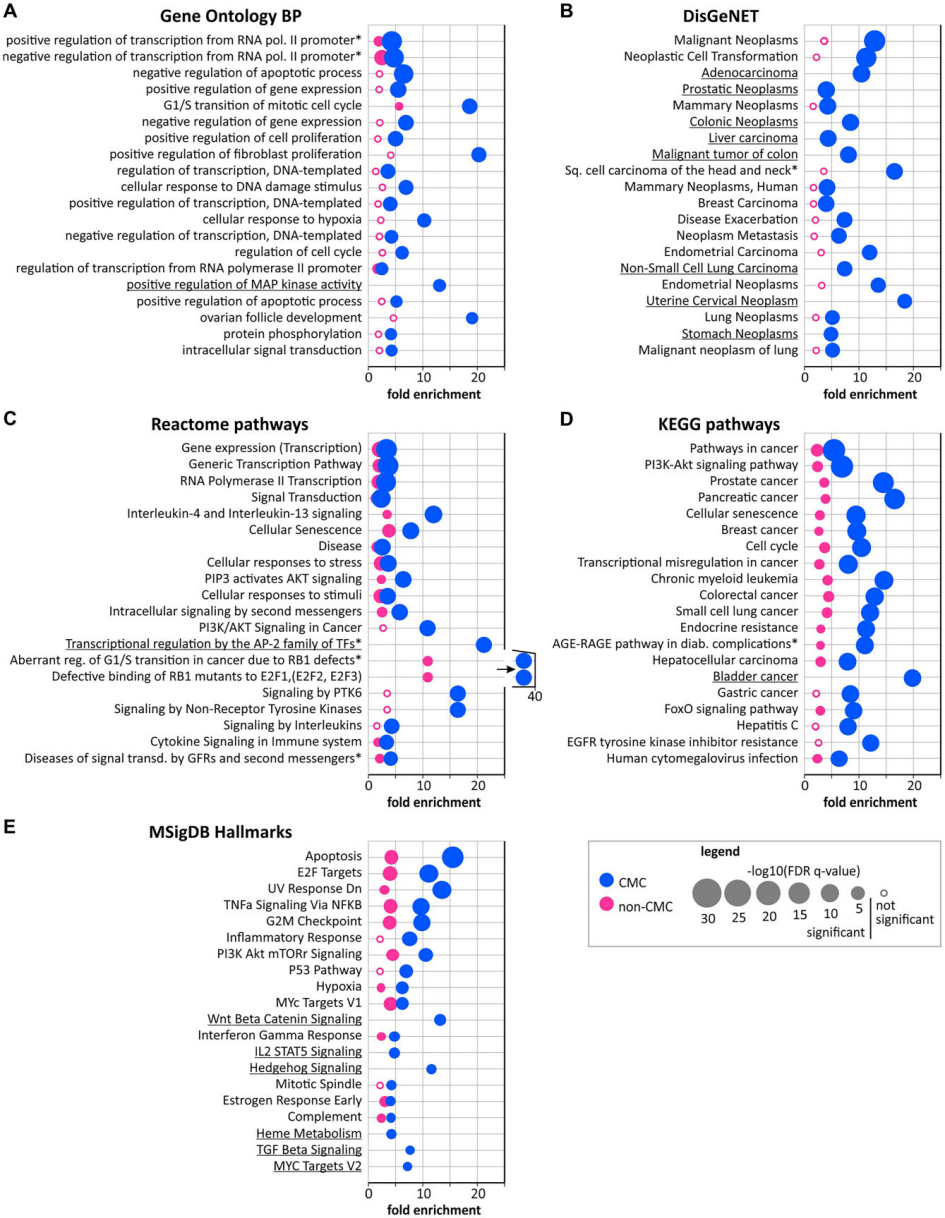
Association of CMC and non-CMC target genes with different functional categories. (A–E) Bubble plots illustrating FE of CMC (blue bubbles) and non-CMC (pink bubbles) target genes in gene categories defined by Gene Ontology BP terms (**A**), DisGeNET-associated diseases (**B**), Reactome pathways (**C**), KEGG pathways (**D**) and MSigDB hallmarks (**E**). Bubble sizes are proportional to –log_10_(*q*-values) (see legend). Each plot shows 20 representative categories associated with CMC miRNA gene targets with the lowest *q*-values. Full lists of associated categories are shown in Supplementary Table S8. Asterisk indicates terms with shortened names presented on the plot; for underlined terms no enrichment was indicated in non-CMC.

In total, the analysis yielded 103 and 4 Gene Ontology BP terms, 193 and 0 DisGeNET defined diseases, 106 and 72 Reactome pathways, 103 and 40 KEGG pathways, and 22 and 15 MSigDB hallmarks significantly enriched, respectively, in CMC and non-CMC target genes (Supplementary Table S8). Noticeably, as shown in Supplementary Table S8 and Figure 4, most terms associated with CMC (especially the top-associated ones) are either directly related to cancer or to processes playing a role in cancer. For example, among the CMC-associated categories are Gene Ontology BP terms related to the regulation of transcription, gene expression, cell proliferation, apoptosis, cell cycle and hypoxia; DisGeNET diseases almost exclusively defined as neoplasms and carcinomas; Reactome pathways related to transcription, cell cycle, and signal transduction, including ‘Interleukin-4 and interleukin-13 signaling’, ‘PIP3 activates AKT signaling’, ‘PI3K/AKT Signaling in Cancer’ and ‘Signaling by PTK6’; KEGG pathways such as ‘Pathways in cancer’, ‘PI3K-Akt signaling pathway’, ‘Transcriptional misregulation in cancer’ and ‘FoxO signaling’, and pathways related to specific cancers such as ’Prostate cancer’, ’Pancreatic cancer’ and ’Breast cancer’; and MSigDB hallmarks such as ‘Apoptosis’, ‘E2F Targets’, ‘TNFa Signaling Via NFKB’, ‘G2M Checkpoint’, ‘PI3K Akt mTOR Signaling’, ‘P53 Pathway’ and ‘Hypoxia’. Non-CMC target genes are also enriched in some of the cancer-related categories, however, their associations show substantially lower FE values and higher q-values. Many of these associations are not significant or only borderline significant (Figure 4 and Supplementary Table S8). Also contrary to CMC, non-CMC target genes are often associated with clearly non-cancer-related categories, such as ‘Global developmental delay’, ‘Intellectual Disability’ or ‘Generalized hypotonia’ defined in DisGeNET (Supplementary Table S8).

Finally, taking advantage of the selected lists of CMC and non-CMC-associated target genes, we compared them with the CGC genes, namely, the list of genes causally implicated in cancer (26). Of the 181 unique CMC target genes, 44 (24%) were annotated in CGC, while only 56 (10%) of 576 non-CMC target genes were in CGC (FE = 1.84, *P*= 2E-6), confirming the functional association of CMC miRNA genes with cancer.

### Classification of CMC miRNA genes as potential oncogenes and tumor suppressors

**Figure 5.**
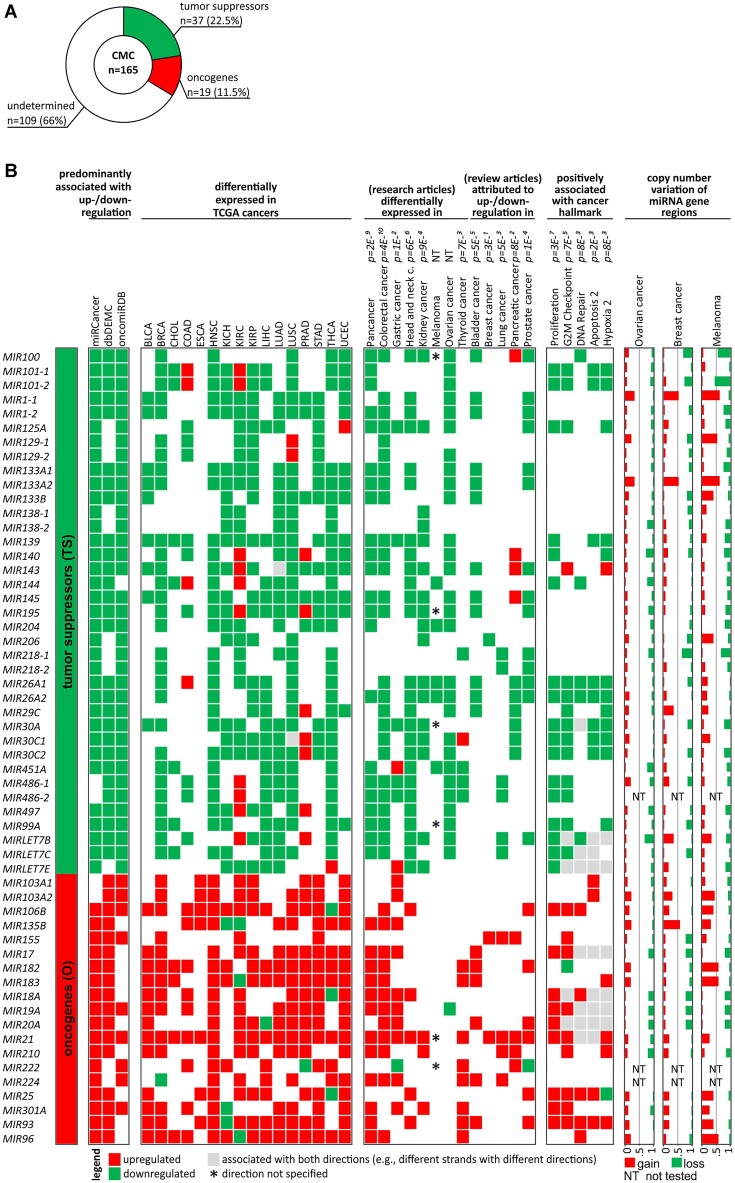
miRNA genes assigned as potential tumor suppressors or potential oncogenes. (**A**) Proportion (number and percent) of miRNA genes assigned as tumor suppressors, oncogenes, and unassigned to any category (undetermined) out of all (n165) CMC miRNA genes. (**B**) List of potential tumor suppressor and oncogene miRNA genes with their characteristics. From the left: list of miRNA genes; heatmap showing the predominant direction of associations (≥75%) with either decreased level/tumor suppressor properties (green) or increased level/oncogenic properties (red) reported in the miRCancer, dbDEMC, and oncomiRDB databases (indicated above); heatmap showing significant expression level changes in 16 cancer types analyzed in TCGA (based on OncomiR; standard TCGA cancer type abbreviations are indicated above); heatmap showing expression level changes in different cancer types (indicated above) as specified in research and review articles used before for validation of CMC; heat map indicating associations with cancer hallmarks (indicated above; according to (36)) positively associated with the suppressor/oncogene status of miRNA genes; graphs showing the proportion of gains and losses in genomic regions encompassing miRNA genes in three cancer types (specified above; according to (66)).

Taking advantage of the collected data, based on the direction of associations reported in the databases (criteria I to III; see Methods) and the direction of changes in expression level in 16 TCGA cancer types (based on the OncomiR database), 19 of the CMC miRNA genes were classified as potential oncogenes and 37 as potential tumor suppressors (Figure 5 and Supplementary Table S9). One hundred and nine miRNA genes were not classified into any of the above groups due to a lack of sufficient evidence or inconsistent direction of associations. As shown in Figure 5 the classification of miRNA genes to oncogenes and tumor suppressors correlates well with expression changes in different cancer types (reported in the research and review articles used above for validation of the CMC list; as in Figure 3D) and positively correlates with the direction of associations of 5 of the individual cancer hallmarks (Proliferation, G2M Checkpoint, DNA repair, Apoptosis 2, and Hypoxia 2; defined by (36)). Also, as shown in Figure 5, the oncogenes/tumor suppressor miRNA genes undergo frequent copy number alterations in cancers (breast, ovarian, and melanoma; as determined in (66)) which may affect their level in particular cancers. However, it must be noted that as miRNA genes are often located in fragile chromosome sites and copy-number variable regions (66–68), most of these alterations are likely randomly occurring accidental events.

## Discussion

Since the first report of a miRNA-cancer link, i.e. deletion of the tumor suppressor *MIR15A/16-1* cluster in chronic lymphocytic leukemia (CLL) reported by George Calin and Carlo Croce in 2002 (30), interest in the role of miRNAs in cancer has grown remarkably, and numerous associations of various miRNAs with cancer have been identified (69). Nevertheless, there is still no defined objective list of cancer-related miRNA genes that can serve as a reference for various studies of miRNAs in cancer, similar to how CGC is used in studies of protein-coding genes.

Although there are several databases dedicated to collecting different types of data about the association of miRNAs with cancer such as miRCancer, OMCD or CancerMIRNome (22,70,71), none of them distinguish a list of cancer-related miRNAs. Also, such a reference list of cancer-related miRNA genes cannot be easily extracted from the published literature, as considering the extensive number of publications reporting associations of miRNAs with cancer, almost all miRNAs, even the questionable ones, would have to be classified as cancer-related, which would make the list uninformative.

Therefore, here, we present CMC, the first list of 165 cancer-related miRNA genes, the selection of which was based on a scoring system built on several independent criteria taking into account different aspects of potential associations of miRNA genes with cancer. The characteristics of all CMC miRNA genes are summarized in Supplementary Table S9. Among the considered criteria were the numbers of miRNA-cancer associations reported in three independent databases, consistency of reported associations (with either potential oncogenic or tumor suppressor role), assignment of miRNAs to particular cancer hallmarks, or the ‘MicroRNAs in Cancer’ KEGG pathway, and cancer genetic alterations suggesting cancer-driven positive selection of genetic alterations in miRNA genes. Additionally, based on the collected data, we classified 19 and 37 of the CMC miRNA genes as either oncogenes or tumor suppressors, respectively. As shown in Figure 5, the assignment of miRNA genes to oncogenes and tumor suppressors is generally associated with increased and decreased levels of the corresponding miRNAs in cancer and positively associated with expression signatures of five of the tested cancer hallmarks (Proliferation, G2M Checkpoint, DNA repair, Apoptosis 2, and Hypoxia 2; defined by (36)), which confirms the relevance of the assigned categories. Also, the observed excess of tumor suppressors over oncogenes is consistent with the general notion that miRNAs play mostly tumor suppressor roles and that generally the global miRNA level is decreased in cancer, the phenomenon first reported by Lu *et al.* in 2005 (72) and subsequently confirmed in multiple studies (73,74). Considering however the pleiotropic character of many miRNA genes regulating multiple targets, some of the CMC miRNA genes still may have distinct functions in specific cancer types or in specific conditions and may play a role in other non-cancer diseases, for example, *MIR34A*, which plays an important role in cancer also plays a role in neurodegenerative diseases and regulation of the immune system (75,76).

We believe that CMC may be useful for verifying the reliability of miRNAs identified in different types of cancer analyses (especially whole miRNome analyses such as differential expression profiling) or optimizing statistical procedures to identify cancer-driver miRNA genes based on analysis of cancer somatic mutations, an increasing number of which have been recently identified in noncoding regions (11,29,77). It may also be a useful reference for any studies of miRNAs in cancer. Based on similar rationales and recognizing a similar lack of reference resources for different classes of noncoding RNAs, Rory Johnson's group recently created a list of cancer-related lncRNAs (CLC) (78). Despite a very short time from the initial release (2020), three versions of CLC have already been published (35,78,79).

The additional advantage of CMC is the scoring system that enables adjustment of the list of cancer-related miRNA genes to individual needs, which allows additional weighting of the observed associations, and what is especially important, which allows further upgrading of CMC with the use of new criteria or correction of existing criteria.

We have validated the credibility and robustness of CMC in various ways, including leave-one-out analyses, comparison with other resources of miRNA-cancer associations, and analysis of miRNA targets enrichment in biological pathways and processes. An important part of the validation analyses was the comparison of CMC with miRNA genes impacting the growth and fitness of cancer cells, identified using CRISPR-based miRNA gene knock-out screenings (52,53), which showed enrichment of the identified miRNA genes in CMC (Figure 3F). As CRISPR knock-out screening is an unbiased tool that links loss-of-function alterations in tested genetic elements to a phenotype of interest, the results of these comparisons are completely independent of other miRNA gene analyses, their expression levels (detectability), and the popularity of particular miRNA testing. Additional confirmation of the validity of the CMC scoring system is the fact that among miRNA genes with the highest scores are the genes of the quintessential cancer-related miRNAs (3,4). For example, the top miRNA gene in CMC is *MIR21* with a maximum possible score of 7. Encoded by *MIR21*, miR-21-5p is arguably the best-known oncogenic miRNA. Its level is increased in many types of cancers, e.g. colorectal, breast and lung cancers. It promotes cell proliferation, migration, and invasion and inhibits apoptosis by downregulating such targets as *PDCD4* (programmed cell death protein 4) and *PTEN* (phosphatase and tensin homolog) (80). One of the top miRNA genes, with a score of 6 points, is *MIR16-1*, which together with *MIR15A* (also with a high score in CMC) constitutes the above-mentioned *MIR15A/16-1* cluster (also known as the *DLEU2* – *deleted in lymphocytic leukemia 2* locus), the first identified tumor suppressor miRNA locus deleted in >50% of cases of B-cell CLL (30,81,82). In addition to CLL, decreased expression of *MIR16-1*, as well as *MIR15A*, has been observed in multiple cancers, including breast, lung, colon, ovarian, and prostate cancer (83–87). The most important target of the *MIR15A/16-1* cluster is the oncogene *BCL2*, with a role in promoting survival and inhibiting cell apoptosis (88). The loss of the *MIR15A/16-1* cluster due to 13q14 deletion has been shown to be the main reason for *BCL2* overexpression in CLL (89,90). On the other hand, the first described oncogene miRNA locus is the *miR-17-92a-1* cluster, which consists of 6 miRNA genes, i.e. *MIR17*, *MIR18A*, *MIR19A*, *MIR20A, MIR19B-1* and *MIR92A-1*, all of which are in CMC with high CMC scores (Figure 2, Supplementary Table S4). It was initially identified as a target of 13q31-32 amplification in diffuse large B-cell lymphomas (DLBCL) (91), but later it was found amplified and overexpressed in other hematological and solid cancers, including medulloblastoma, lung, and colon cancer (92–95). The expression of miRNAs derived from the *miR-17-92a-1* cluster has been shown to promote cell proliferation, suppress apoptosis and induce angiogenesis (reviewed in (95)). Other genes of well-documented cancer-associated miRNAs with high CMC scores include *MIR155* (score of 5.5), *MIR205* (score of 5), and *MIRLET7A1* (score of 4.5) [summarized in (3,4)]. Another informal confirmation of the validity of the CMC scoring system may be the fact that all miRNA genes whose miRNAs have been tested in clinical trials of cancer therapies either as anti-cancer drugs (miRNA mimics, i.e. *MIR16-1*, *MIR34A* and *MIR193A*) or targets for miRNA inhibitors (antagomiRs; i.e. *MIR155* and *MIR10B*) (96) are included in the CMC, mainly with very high CMC scores (Figure 2, Supplementary Table S9). For example, the first miRNA therapeutic tested in a clinical trial was MRX34, a double-stranded mimic of miR-34a-5p (ClinicalTrials.gov: NCT01829971). Although the trial had to be terminated due to serious immune-related side effects, the treatment succeeded in downregulation of the miR-34a-5p oncogenic targets, including genes playing a role in cancer immune evasion (97,98). Additionally, many CMC miRNA genes (e.g. *MIR21*, *MIR124*, *MIR141*, *MIR155*, *MIR375*, *MIRLET7A*) encode miRNAs being tested as cancer biomarkers (see references (80,96)). As a specific example, a panel of 6 miRNAs, all encoded by the CMC miRNA genes (*MIR21*, *MIR20A*, *MIR103A, MIR106B*, *MIR143* and *MIR215*), whose profile (score calculated based on miRNA levels) is being clinically tested as a biomarker for efficacy of adjuvant therapy in stage II colon cancer (ClinicalTrials.gov: NCT02466113).

Despite numerous associations of CMC with various characteristics of cancer, CMC is not free of limitations that should be considered while using it. Probably the most serious issue is ascertainment bias resulting from the fact that some miRNAs (e.g. more highly expressed, more conserved, or earlier identified) are more frequently studied than others and therefore more frequently detected or included in further analyses. To mitigate this issue, we excluded from consideration low-confidence miRNAs, focusing our analysis only on miRNA genes validated by either miRBase or MirGeneDB, which should be considered as a background miRNA list while using CMC. Second, the CMC cutoff score was set in such a way as to exclude false positives in the first place. This conservative threshold, although justified by leave-one-out analysis, is likely to exclude some genuine cancer-related miRNA genes currently scored in a gray zone (CMC score < 2). However, with the increasing amount of data and refinement of the CMC score, some of the gray zone miRNA genes may be reclassified in further versions of CMC. Third, it is very likely that there are still miRNA genes that are not yet annotated or are not sufficiently validated to be included in our analysis. It is estimated that a complete list of human miRNA genes may equal even ∼2000, a substantial number of which are not annotated in the current version of miRNA databases; on the other hand, many annotated miRNAs are of low credibility (14,18,99). Fourth, as mentioned above in the Results section, many studies reporting miRNA–cancer associations are of low reliability, on the one hand, increasing the ascertainment bias, and on the other hand, reporting false-positive associations. Finally, in the current version, CMC does not specify associations of miRNA genes to specific cancer types. Although it is known that some miRNAs are tissue-specific and are associated predominantly with specific cancer types, for example, *MIR122* with liver cancer (100), or *MIR142* with hematological cancers (101), at present, we do not have adequate data to reliably assign a significant proportion of miRNA genes to specific types of cancer. As discussed above, the current version of CMC is not a finite list of cancer miRNA genes and as new data becomes available, it may be updated. New data may be added to the CMC score, either by modifying existing criteria or as new criteria. As a result, some (especially borderline) miRNA genes might be reclassified, i.e. added or removed from CMC. On the other hand, considering the robustness of the CMC, the main body of it will most likely remain immutable. We expect, that due to the growing popularity of whole-genome sequencing and analysis of non-coding regions, a criterion that may gain importance/weight in the near future is criterion VII ‘genetic evidence’. Also, CRISPR knockout results may become an important source of information, potentially in the future considered as a new CMC criterion.

In summary, we provide here CMC, the first comprehensive reference list of cancer-related miRNA genes. The list was constructed based on several independent criteria and was validated by comparisons with various cancer data. The CMC may be used as a reference for the validation of results of miRNA analyses in cancer, for prioritization of miRNAs in different cancer analyses, and for optimization of methods for identifying further cancer-related miRNAs or cancer-driver miRNA genes. It may also help in classifying the knowledge regarding the role of miRNAs in cancer.

## Supplementary Material

gkae017_Supplemental_Files

## Data Availability

The data underlying this article are available in the article and in its online Supplementary material. All resources and datasets used in this study are publicly available and can be accessed as indicated in the paper.
