# Supplementary material for: CMC: Cancer miRNA Census – a list of cancer-related miRNA genes
Source: Nucleic Acids Res. 2024 Jan 23;52(4):1628–44. doi: 10.1093/nar/gkae017 (PMC10899758; doi:10.1093/nar/gkae017)
Supplement: gkae017_Supplemental_Files [file gkae017_supplemental_files.zip › 231122_SuszynskaM_Supplementary_Figures.pdf]

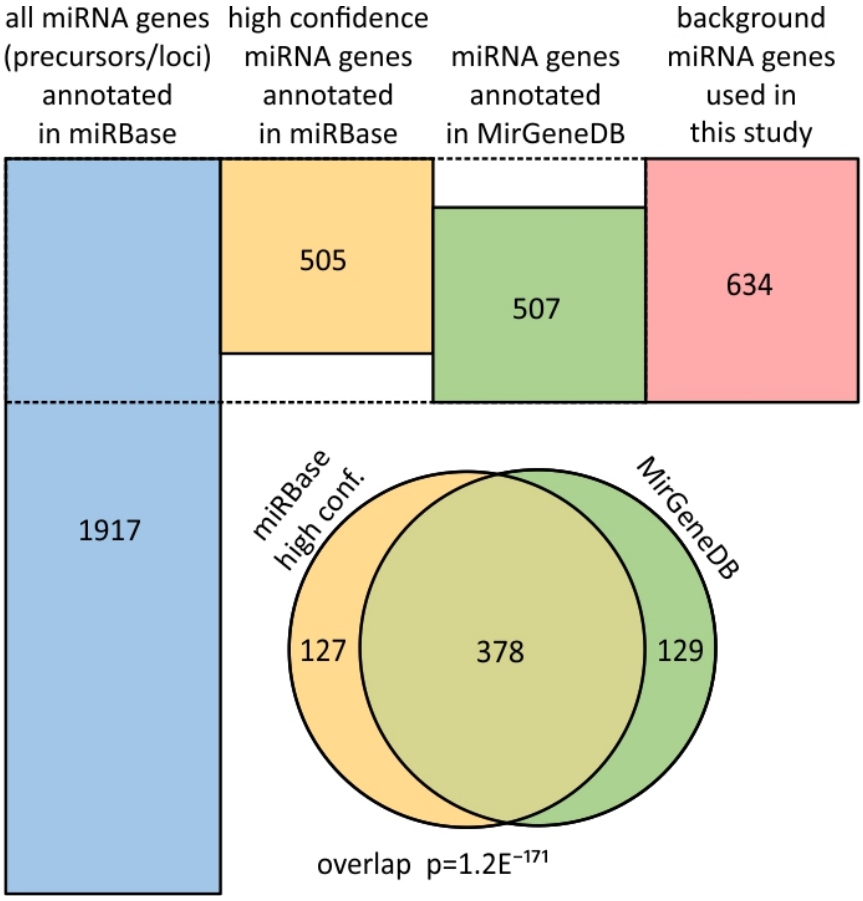


**Supplementary Figure S1. Selection of background miRNA genes used in this study.** The subsequent columns depict the proportion (number) and overlap of the following group of miRNA genes: total miRNA genes in miRBase (blue color), miRNA genes designated as ‘high confidence’ in miRBase (yellow color), miRNA genes annotated in MirGeneDB (green color), and miRNA genes used in this study as background miRNA genes (salmon color). The area-proportional Venn diagram (created with the BioVenn web tool; in inset) depicts the overlap between miRNA genes designated as ‘high confidence’ in miRBase and miRNA genes annotated in MirGeneDB, cumulatively constituting the background miRNA genes.

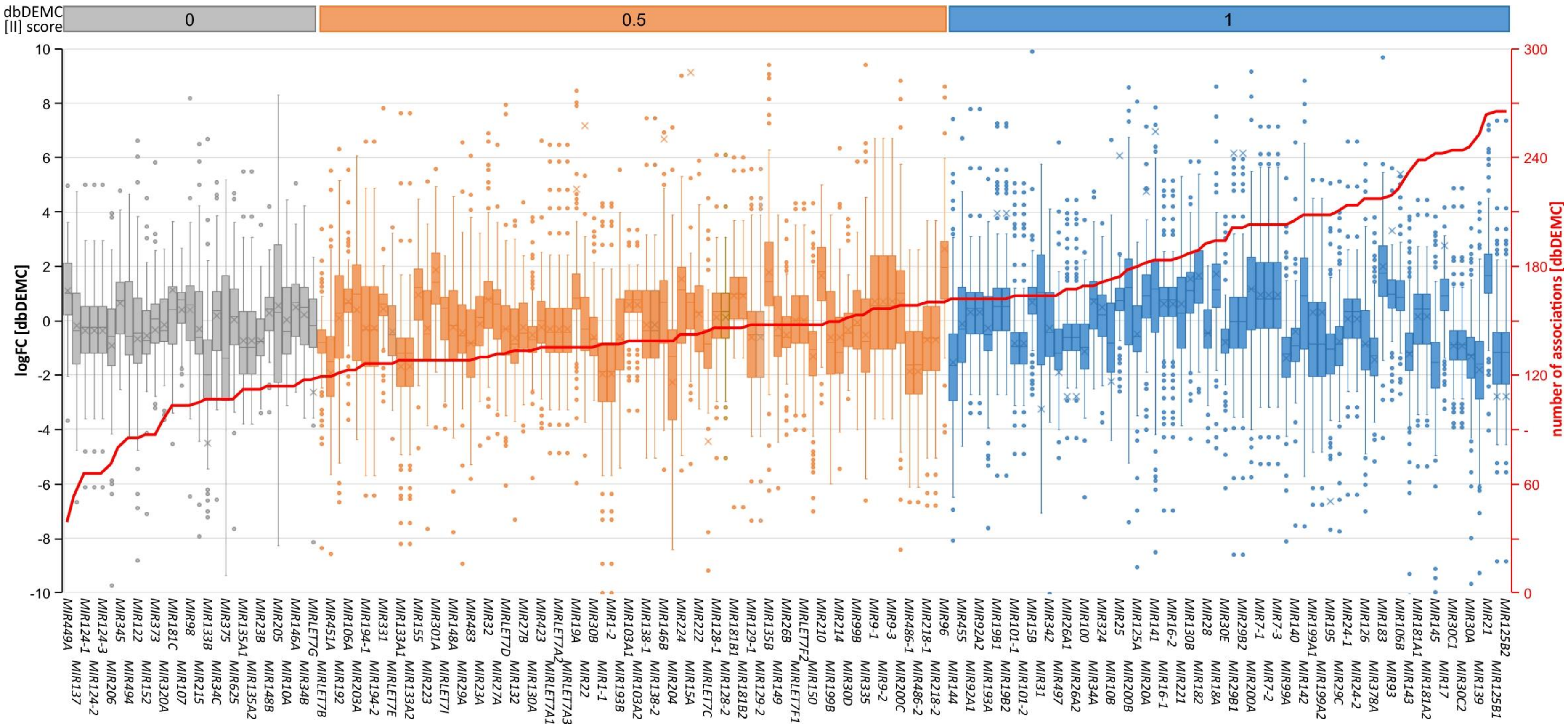


**Supplementary Figure S2. Differential expression logFC values representing miRNA-cancer associations detected/deposited in dbDEMC for the CMC miRNA genes.** The miRNA genes were sorted according to the increasing number of detected associations (x-axis) and divided into 3 categories with 0, 0.5, and 1 points assigned in criterion II. In each box-and-whisker plot vertical lines in the box represent the lower quartile (25th percentile), median (50th percentile), and upper quartile (75th percentile) of significant miRNA-cancer associations; whiskers indicate the highest and lowest values within 1.5 interquartile range; mark "x" shows mean; and the dots indicate outliers. The red line indicates number of associations.

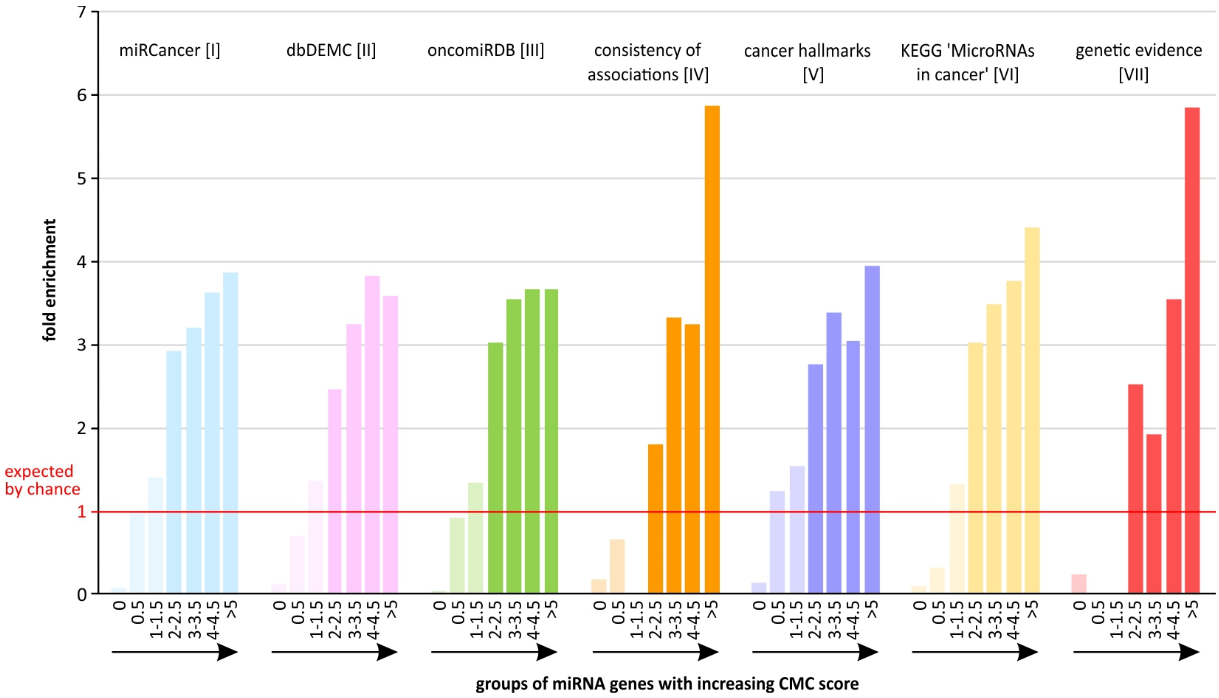


**Supplementary Figure S3. Evaluation of the optimal CMC/non-CMC miRNA gene threshold using leave-one-out analysis.** Bar plot showing FE values (y-axis) of miRNA genes positively scored in criteria I-VII (indicated above) in groups of miRNA genes with the following CMC scores: 0 points, 0.5 points, 1-1.5 points, 2-2.5 points, 3-3.5 points, 4-4.5 points, and ≥5 points (x-axis). FE values for a particular criterion were calculated after excluding this criterion from CMC scoring (see the leave-one-out procedure in Materials and Methods). Pale-color and bright-color bars indicate the miRNA gene groups below and above the CMC threshold assumed in the study (≥2), respectively. The red horizontal line indicates the FE level (=1) expected by chance.
